# IoT-Based Emergency Vehicle Services in Intelligent Transportation System

**DOI:** 10.3390/s23115324

**Published:** 2023-06-04

**Authors:** Abdullahi Chowdhury, Shahriar Kaisar, Mahbub E. Khoda, Ranesh Naha, Mohammad Ali Khoshkholghi, Mahdi Aiash

**Affiliations:** 1 School of Computer Science, University of Adelaide, Adelaide 5005, Australia; 2 Department of Information Systems and Business Analytics, RMIT University, Melbourne 3000, Australia; shahriar.kaisar@rmit.edu.au; 3 Internet Commerce Security Laboratory, Federation University Australia, Mount Helen 3350, Australia; m.khoda@federation.edu.au; 4 School of ICT, University of Tasmania, Hobart 7005, Australia; 5 Centre for Smart Analytics, Federation University Australia, Churchill 3842, Australia; 6 Department of Computer Science, Middlesex University, London NW4 4BT, UK; a.khoshkholghi@mdx.ac.uk (M.A.K.); m.aiash@mdx.ac.uk (M.A.)

**Keywords:** intelligent transportation system, emergency vehicle priority, drone in emergency

## Abstract

Emergency Management System (EMS) is an important component of Intelligent transportation systems, and its primary objective is to send Emergency Vehicles (EVs) to the location of a reported incident. However, the increasing traffic in urban areas, especially during peak hours, results in the delayed arrival of EVs in many cases, which ultimately leads to higher fatality rates, increased property damage, and higher road congestion. Existing literature addressed this issue by giving higher priority to EVs while traveling to an incident place by changing traffic signals (e.g., making the signals green) on their travel path. A few works have also attempted to find the best route for an EV using traffic information (e.g., number of vehicles, flow rate, and clearance time) at the beginning of the journey. However, these works did not consider congestion or disruption faced by other non-emergency vehicles adjacent to the EV travel path. The selected travel paths are also static and do not consider changing traffic parameters while EVs are en route. To address these issues, this article proposes an Unmanned Aerial Vehicle (UAV) guided priority-based incident management system to assist EVs in obtaining a better clearance time in intersections and thus achieve a lower response time. The proposed model also considers disruption faced by other surrounding non-emergency vehicles adjacent to the EVs’ travel path and selects an optimal solution by controlling the traffic signal phase time to ensure that EVs can reach the incident place on time while causing minimal disruption to other on-road vehicles. Simulation results indicate that the proposed model achieves an 8% lower response time for EVs while the clearance time surrounding the incident place is improved by 12%.

## 1. Introduction

Worldwide adoption and use of intelligent transportation systems (ITS) have improved traffic and safety conditions, reduced traffic congestion, and the timely propagation of important traffic updates. ITS uses special hardware (e.g., inductive loop detectors, radar detectors, and laser detectors) and roadside infrastructures to evaluate existing traffic conditions (e.g., traffic flow at a specific point of the road system, average travel times for a particular segment of track, and road hazard). Real-time traffic monitoring capability and the improved communication facility between vehicle and everything (V2X) have enabled an ITS to offer various services, such as dynamic traffic signal control, emergency vehicle priority, public vehicle priority, pedestrian safety, incident management service, variable speed limit, and freeway ramp signals. An emergency management system (EMS) is a crucial component of ITS, which detects emergencies (e.g., road accidents or fires) and automatically send emergency vehicles (EVs) (e.g., ambulances, fire service, and police) to appropriate locations.

Emergency services are typically available in most major cities, and although their goal response time and the quality of service (QoS) requirements are strict, congestion can severely impede these services. For instance, the average real response time for Victoria, Australia, is 17% higher than the target of 90th percentile [1], and the standard set by the US National Fire Protection Association (NFPA) suggested eight minutes target time for EVs [2]. EVs (e.g., ambulances, fire service, and police) need to arrive at the location of an emergency, such as an accident or fire, as soon as possible, specifically within the “golden time”, which is an early critical time to rescue people in an incident during which damage attributable to the incident can be minimized. For example, in first aid, cardiopulmonary resuscitation (CPR) should be commenced within 5 to 10 min from the time a situation occurs. Similarly, the amount of damage may vary significantly based on the time firefighting begins after a fire occurs. In most countries, providing giveaways to EVs is a legal requirement. The drivers of regular (i.e., non-emergency) vehicles are expected to pull over or change lanes or give way to the EVs as soon as possible when they hear the siren or see the emergency lights. However, on urban roads, specifically during peak congestion hours, it becomes hard and sometimes impossible for a driver to change lanes or give way to EVs due to the presence of other vehicles. This results in the late arrival of EVs and causes increased fatalities and social expenses [3].

Researchers have addressed the above-mentioned issues and proposed various techniques to improve EV response time. Hannoun et al. [4] suggested that nonpriority vehicles should pull over when there is any EV behind them. This solution is feasible for the highway or freeway with multiple lanes. However, this pull-over method will cause significant delays due to average speed loss due to the introduction of oscillations and capacity drop for the stop-and-go process. A few other works [5,6,7] suggested that the traffic signals ahead of the emergency vehicles should be changed to green using RFID sensors, inroad sensors, and EV detection from a video feed. Although these methods can slightly improve the EV travel time, they do not consider disruption or congestion faced by other nonemergency vehicles adjacent to the EVs’ travel path. This aspect is significantly important to reduce the congestion near the incident place and improve the incident clearance time. Incident clearance time refers to the time from the start of the incident to bring the traffic condition back to the preincident traffic condition. Existing approaches also did not consider the real-time traffic monitoring capability of ITS and the communication features of its roadside infrastructure. They connected autonomous vehicles, which can aid in optimal travel path selection for EVs based on real-time traffic conditions. Furthermore, the speed loss of EVs near the intersection for safety reasons is not addressed in existing methods.

To address the above-mentioned issues, we propose an integrated UAV-assisted emergency vehicle priority system in this paper. Our proposed approach considers that the EMS can automatically detect an incident type (e.g., road accident or fire) and place and, based on the type of the incident, send appropriate EVs. In this case, EMS communicates with the central traffic controller to obtain an optimal travel path for sending EVs. In response, the central traffic controller utilizes the ITS sensors to determine the quickest EV travel path based on the real-time traffic condition and informs the EMS, who delivers this information to EVs.

The proposed model also uses an adaptive path selection strategy that continues monitoring the traffic condition while the EVs are en route and notifies them immediately when a better route becomes available. Drone assistance near the intersection to reduce the speed loss of EVs is also utilized in our proposed model to improve performance.

To assess the performance of the proposed model, we used extensive simulation using the Simulation of Urban Mobility (SUMO) version 1.17.0 [8] for macOS and real-life traffic data from VicRoads—the road corporation of Victoria, Australia [9]. The simulation results show that our proposed approach yielded better results than existing methods. Overall, this paper makes the following contributions:

1. Developed an adaptive optimal travel path selection method for EVs considering real-time traffic conditions and wait time of surrounding nonemergency vehicles.
2. Proposed using drones as the lead vehicle of EVs to minimize their speed loss near intersections while improving safety conditions by notifying other nonemergency vehicles about incoming EVs.
3. Conducted extensive simulations based on real-life traffic and incident datasets obtained from VicRoads, and the simulation results demonstrated the superiority of our proposed model compared to existing approaches.

The rest of the paper is organized as follows: In Section 2, we describe the issues related to incident management in ITS, the use of drones in different sectors, and current emergency vehicle priority systems. The proposed model is discussed in Section 3. In Section 4, we analyse the performance of the proposed model. Finally, Section 5 concludes the paper.

## 2. Background

Countries and organizations worldwide are making strides to improve road safety and workplace safety [10], introducing various measures from stricter traffic laws to more advanced traffic management systems. Despite these commendable efforts, the number of incidents on roads is on the rise [11,12,13].

Emergency Management System (EMS) provides different services, such as monitoring the number of vehicles and their speed on the road, measuring traffic flow rate, and managing traffic incidents. EMS aims to reduce traffic congestion and disruption faced by road users while minimizing the incident clearance time. One of the major challenges in minimizing incident clearance time is to send the EV to the incident place within the shortest possible time. Some highways and freeways have dedicated emergency lanes, and the EVs can use those. However, on arterial urban roads, mainly during the peak period, EVs must stay behind other nonemergency vehicles.

Clearing the incident place is one of the primary objectives of EMS, and several management teams work together to achieve this [14]. Existing literature used a few approaches, such as Green Wave [5], ITS Integration model [15], Usage-Base model [16], Smart Congestion Avoidance (STLC) [17] and Smart Collision Avoidance (SCA) [18] to aid emergency service vehicles to travel quickly. The Green Wave system [5] recommended making one signal green in front of an EV using RFID, wireless sensors, and video cameras. In the ITS integration model [15], different roadside infrastructures (e.g., traffic signal, traffic controller) communicate together to provide priority to EVs. The usage-Base model [16] ensures the best utilization of the EV department’s resources (e.g., Staff, number of available EVs). In contrast, STLC [17] provides EVs with a better route to avoid the possible congested route, while the SCA [18] system uses different warning signs to ensure on-road vehicles can avoid collisions, mainly at the intersections. There are many emergency vehicle priority systems already implemented in different countries, such as Intelligent Traffic Light System in Israel [19], the Smart Traffic Signal System in Singapore [20], the GPS-based emergency vehicle priority in Chicago, USA, and the Emergency Vehicle priority system in Queensland, Australia [21].

Qin and Khan [22] proposed a preemption method for EVs that allows the central traffic controller to set an emergency traffic phase so that the signal phase in front of an EV turns into a green signal. On the other hand, Huang et al. [23] developed a Petri-net-based preemption model, which divided the EV travel route into different discrete subsystems. In this method, the central traffic control system sets priorities for different sections of the incident place. The higher priority area gets a higher green phase time so EVs can reach the incident place quicker. Erskine and Elleithy [24] proposed a joint strategy of reducing the wait time for an EV by choosing the best path selection strategy and minimizing the negative impact of EV preemption on general traffic. However, this approach shows severe negative consequences in the case of large traffic volumes where the spacing of the intersections is shorter compared to the minimum detection distance. This approach is also ineffectual when multiple EVs follow each other as the recovery phase is not designed to explicitly handle the preemption of the 2nd EV. The above-mentioned models also did not calculate the impact of the preemption policy on adjacent nonemergency vehicles.

To detect the presence of an EV in an intersection, different authors proposed roadside cameras [25], EV GPS-based location [26], and RFID tags [27]. In [28,29], the authors proposed using vehicular and wireless sensor networks to determine the location of the EV. These methods used different localization-based algorithms [30,31,32] to provide a green signal ahead of an EV. Although the methods mentioned above were able to reduce the travel time of an EV to the incident place, they only considered turning only one signal green ahead of the EV. In contrast, Emergency Vehicle Priority System (EVPS) [33] assigned different priority levels to different EVs based on the type of incident. In this case, the optimal number of signals that needed to be turned green was calculated considering the impact of EV’s travel path on adjacent nonemergency vehicles. Although this approach achieved improved performance in EV travel time and incident clearance time, this did not consider the increase in wait time of other nonemergency vehicles.

Amini et al. [34] considered human-driven vehicles along with connected vehicles (Internet of Vehicle environment) to observe the impact of vehicle speed on different driving behaviors. They also tested the vehicle speed change caused by different phase times, the impact of sudden brakes by leading vehicles, and the speed change near an intersection. The result shows that the change in speed of a leading vehicle has a huge impact on the following vehicle. In [35], the authors proposed a signal control setting by providing a split queue to the vehicles in an intersection to minimize the delay in crossing an intersection. This method needs multiple phases in an intersection to work. However, no prior works have considered maintaining a better average travel speed of EV while entering an intersection.

Unmanned Aerial Vehicles (UAVs) or drones have been utilized in many scenarios, including monitoring public events, rescue tasks, disaster monitoring, and resource distributions [36]. Important concerns for emergency coordination and crisis management strategies are effective deployment, instant availability, and reliability [37]. Drones ensure the efficiency of disaster response and transmission of critical incident reports [38].

UAVs have great potential to enhance emergency response times by coordinating with traffic controllers and directing emergency vehicles along optimized paths. However, their use comes with several constraints. The battery life of UAVs is a significant concern; most commercial drones can only remain airborne for around 20–40 min. This time can also vary depending on the size and weight of the attached equipment (e.g., camera, lights) and weather conditions. These battery life issues significantly impact their operation range and duration, particularly in larger cities or during extended emergencies. UAVs face difficulties operating effectively in adverse weather conditions, such as high winds, heavy rain, snow, and foggy visibility. Communication latency, or delays in data transmission between UAVs and control centers, can compromise the drones’ efficacy in real-time traffic management. For UAV-guided traffic management to be effective, it must be integrated with other traffic and emergency service systems. Not all arterial roads may be covered by UAV-assisted emergency vehicle support systems.

Existing visual roadside units such as speed cameras, red light cameras, and road safety cameras used in existing incident management systems (IMSs) will continue supporting the IMSs [14,33,39,40] when UAV support isn’t available. These RSUs also work as early warning systems by detecting and reporting accidents or other incidents in real-time. This assists in quicker emergency response and more efficient traffic management.

Location awareness is another important feature in responding to emergency incidents. Real-time incident monitoring using a drone (UAV) incorporating sensors’ RF signals, ultrasound, and GPS tracking is used to identify obstacles in [41]. An Intelligent Video Sensor (IVS) integrates image analysis with video sensing and transmission capabilities. It works in [42], suggesting using such embedded systems to collect streaming video, calculate data associated with high-level traffic parameters, and transfer the live video feed and computed traffic data to a base station. Traffic parameters calculated in this work include vehicle flow, average vehicle speed, obstacle detection, and deadlock. Li [43] considered that UAVs can be equipped with two sets of LiDAR where each set can measure the speed of vehicles in one lane, and hence both sets can be used together to measure the speed in multiple lanes. The accuracy of this method was reported as 94–100%. Apeltauer et al. [44] considered that video recordings from intersections can be captured using cameras attached to UAVs, and the recording can be analyzed using classifiers. Similar video analysis-based traffic monitoring is also proposed in [45]. Ke et al. [46] proposed a real-time traffic flow parameter estimation from aerial videos using a four-step process with Kanade–Lucas–Tomasi (KLT) tracker, *k*-means clustering, connected graphs, and traffic flow theory. UAVs have also been used for pedestrian movement monitoring [47]. Recently, Oubbati et al. [48] proposed an exciting work where drones were used to monitor urban areas for incident detection and traffic parameters estimation to help plan a route for emergency vehicles. However, none of the previous works used drones as lead vehicles for EVs to minimize their speed loss while crossing an intersection and to provide better safety for other road users by alerting them about upcoming EVs. Table 1 highlights specifications of a few commercially available common drones, suggesting that most of them offer a communication range of more than 4 km, a flying time of at least 20 min, and are available within an acceptable price, and hence are suitable to assist in incident management.

To the best of our knowledge, no prior work in the literature used UAVs to guide the emergency vehicle to the incident location. A few works [43,46,48] in the literature employed UAVs to estimate traffic parameters, but they did not use UAV assistance to guide emergency vehicles. The goal of the above-mentioned works is to accurately estimate traffic parameters, which is significantly different from our work. Therefore, no direct comparison is possible. However, there are some existing works that focus on assisting emergency vehicles to reach the incident location by using manual intervention on the traffic light system (e.g., changing the green signal phase). We have compared our work with existing incident management systems. In summary, although a few existing works have considered emergency vehicle priority and change of traffic signals ahead of an emergency vehicle to ensure their early arrival, they lack consideration of other important aspects, such as appropriate consideration of disruption faced by other nonemergency vehicles in adjacent areas of the incident place, the dynamic estimation and calculation of traffic parameters, and use of UAVs to ensure better road safety for all road users. Our proposed approach addresses these issues and proposes a UAV-assisted incident management system.

## 3. Proposed Model

### 3.1. Problem Scenario

**Figure 1:**
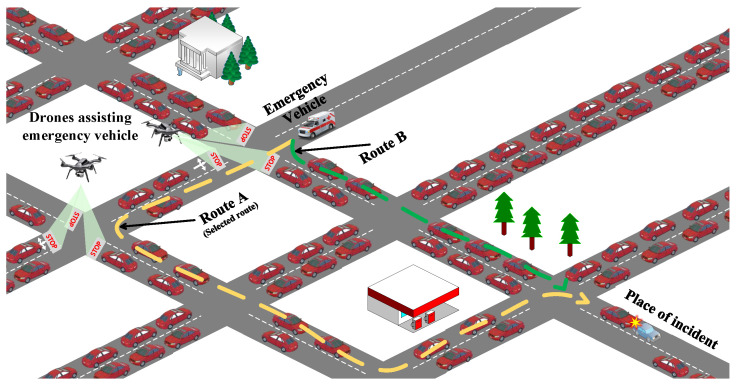
Drone-assisted incident management system. Here, drones will work as the lead vehicles to guide the EVs to the incident place while alerting other road users.

The problem scenario is depicted in Figure 1. The figure shows the current position of the EV that is traveling toward the place of the incident. The task of selecting the best travel path for an EV involves several challenges including:

- Optimising the travel time of the EV
- Reducing incident clearance time
- Minimising disruption faced by other road users.

Figure 1 shows that there are multiple paths available to the place of the incident. However, the path with the shortest distance (Route B) may not be the best path, as it may have higher congestion, or selecting that path may cause a higher disruption for other road users. It is possible to make all the signals green in front of the EV when it starts its journey. Although this may result in faster arrival of EVs, it will also create more congestion for other road users as other nonemergency vehicles traveling in other directions will be stuck in red signals. The problem is further complicated by the dynamic nature of traffic flow and the sudden movement of vehicles. For example, if we consider that a static travel path is selected at the beginning at $t_{0}$ for EVs, this path may not be the best route after five-time units at $t_{5}$ as the traffic condition in that travel path may change completely due to arrival or departure of vehicles. Another problem is while crossing an intersection, EVs need to slow down and reduce their speed to ensure road safety for other road users, and such stop-start movement results in higher travel time for them. These issues are addressed in our proposed model and discussed below.

### 3.2. Proposed Approach

The proposed approach considers that when the emergency controller receives a notification about an incident, it determines the emergency vehicle dispatch location based on the type of incident (e.g., highly critical, medium, noncritical) and the type of the emergency vehicle (e.g., police car, ambulance, fire service) needs to reach the incident location first.

**Figure 2:**
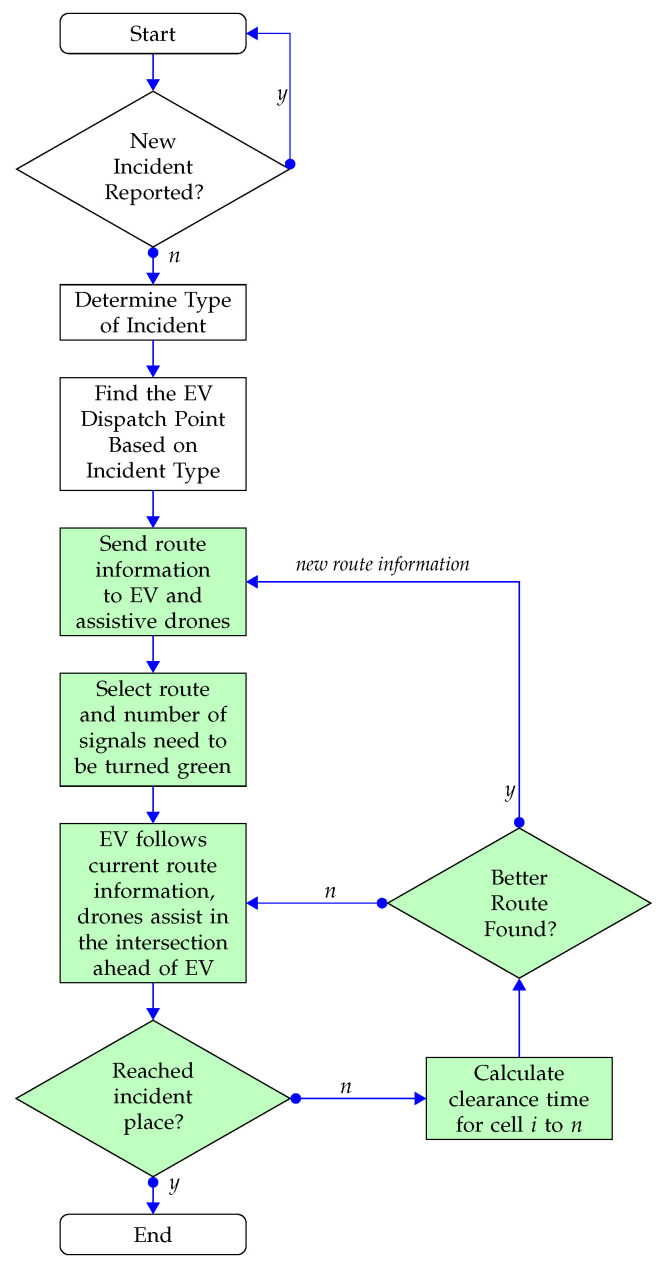
Workflow of UAV-assisted adaptive route selection strategy.

The emergency management system creates various priority codes (for instance, Code A, Code B, Code C) for different incident types. In a Code A scenario, an ambulance is allocated the highest priority (Priority 1), while fire services and police vehicles follow with Priority 2 and Priority 3, in that order. This arrangement is based on the need for an ambulance to be the first respondent at the scene for immediate patient care, followed by the police for incident evaluation. If necessary, fire services are dispatched to deal with potential hazards, such as an oil spill or a fire outbreak. Under Code B circumstances, a fire service vehicle gets top priority (Priority 1) since medical personnel can only assist the injured if the fire is dealt with first. This situation could occur when a vehicle is under fire and occupants are trapped within. For Code C incidents, a police vehicle is given the topmost priority (Priority 1), while the ambulance and fire service vehicles are assigned Priority 2 and Priority 3, respectively. This prioritization is typically for situations that require crowd management, like altercations between groups where a police presence is needed first to disperse the crowd, paving the way for the ambulance to reach the scene. After the emergency vehicle dispatch point and incident place are determined, the emergency control room and emergency vehicle determine the initial route using the emergency vehicle’s GPS [59]. Please note that the above steps are already used in the existing literature [6] and the proposed approach uses the same method. The contribution of the proposed model starts from here and is highlighted in Figure 2. Any new incident in the path of the EV, change of incident (e.g., caught fire when there was no fire before the ambulance started its journey), and any other EV dispatch point is determined by the emergency controller. After an initial travel path for an EV is selected, this information is sent to the central traffic controller, who sends the information to the roadside infrastructure along the travel path and to the assistive drones with a permit to fly permission within the emergency vehicle route.

To ensure the early arrival of an EV, traffic signals along the travel path need to be turned green. A naive approach, in this case, is to turn all the signals green along the travel path of an EV as soon as it starts its journey. Please note that in case of a severe incident and a life-threatening situation this naive approach is used and EVs are provided with the highest levels of priority without any other consideration. However, in other noncritical situations, although this approach will ensure the quicker arrival of an EV, it will also increase traffic congestion for other road users. Therefore, in our proposed approach we optimally determine the number of signals to turn green for the minimum amount of time considering shorter travel time for an EV and reducing the congestion faced by other nonemergency vehicles in adjacent areas.

**Figure 3:**
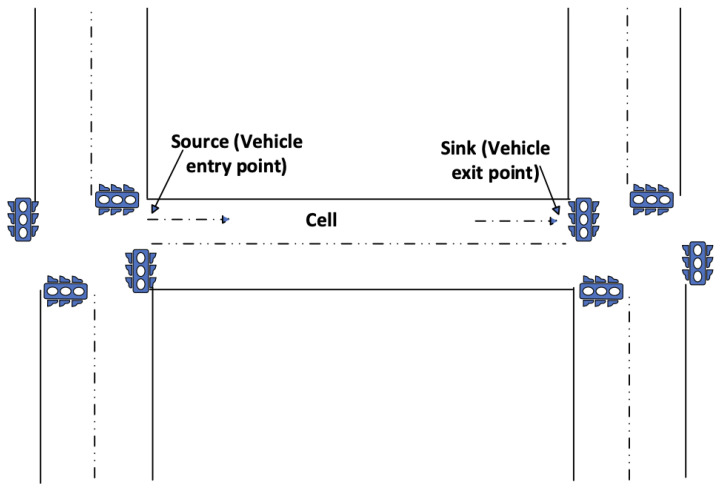
Cell of road with source and sink.

Algorithm 1 outlines the steps followed to determine the number of interruptions (signals need to be turned green) to find of best clearance time. The clearance time here includes the EV travel time to the incident location plus the time needed by other vehicles to clear the cell. In the context of traffic management and transportation research, a “Cell” refers to a distinct section or segment of a road that possesses a single entry point and a single exit point. Roads are divided into cells, where each cell represents a road segment with a designated vehicle entry point (referred to as the “source”) and an exit point (referred to as the “sink”), and there are no additional entry or exit points within the confines of that particular cell. Researchers have employed both fixed cell sizes [60] and variable cell sizes [61] in traffic queuing models. In our proposed model, we adopt the concept of variable cells in traffic modeling, which entails defining the road segment between two intersections as a cell (Figure 3).

The following steps are used to calculate the number of signals that need to be turned green along the EV travel path:

- Determine EV travel time for each cell (1 to *n*).
- Calculate the EV travel time by making multiple signals green (2 to n−1)
- Calculate the number of vehicles in the adjacent cells if different signals are turned green (the number may increase since they have to give way to the EV).
- Calculate the change of speed of other vehicles.
- Find the optimal number of signals that need to be turned green.

**Algorithm 1** Selecting optimal number of signals to turn green.**Input:** route,adj (route to consider, no. of adjacent cells to consider)**Output:** $best_{m}$ (no. of signals to turn green)1:best_clearance_time←∞                                       // initialise to infinity2:$best_{m}\leftarrow \emptyset$                                                                     // initialise to null3:**for** m← 1 to n−1 **do**4:    $EV_{t}\leftarrow$ 0                                                                    // initialise to zero5:    **for each** cell *i* in route **do**6:        $t_{t}\leftarrow CellClearanceTime\left(i\right)$7:        $EV_{t}\leftarrow EV_{t}+t_{i}$8:    **end for**9:    $AD_{t}\leftarrow 0$ // initialise to zero10:    **for each** cell *i* in route **do**11:        **for** a← 1 to adj **do**12:           $L_{a_{i}}\leftarrow a^{th}$ cell to the left of *i*13:           $R_{a_{i}}\leftarrow a^{th}$ cell to the right of *i*14:           $t_{l}\leftarrow CellClearanceTime\left(L_{a_{i}}\right)$15:           $t_{r}\leftarrow CellClearanceTime\left(R_{a_{i}}\right)$16:           $AD_{t}\leftarrow AD_{t}+t_{l}+t_{r}$17:        **end for**18:    **end for**19:    $clearance_{m}=EV_{t}+AD_{t}$20:    **if** $clearance_{m}<best\_clearance\_time$ **then**21:        $best\_clearance\_time\leftarrow clearance_{m}$22:        $best_{m}\leftarrow m$23:    **end if**24:**end for**25:**return** $best_{m}$26:**function** CellClearanceTime($cell_{id}$)27:    $n_{i}\leftarrow$ no. of vehicles in $cell_{id}$28:    $v_{i}\leftarrow$ avg. speed for $cell_{id}$29:    $S_{i}\leftarrow$ space headway for $cell_{id}$ using eq. ()30:    $T_{i}\leftarrow$ time headway for $cell_{id}$ using eq. ()31:    $t_{i}\leftarrow T_{i}\times n_{i}$                                                             // travel time for $cell_{id}$32:    **return** $t_{i}$33:**end function**

The central traffic controller monitors the traffic condition along an EV’s travel path and calculates the cell clearance time in real-time in our proposed model. When an EV enters a cell, the length of the cell, the number of vehicles in that cell, and their average speed are detected. The travel time of an EV to cross an entire cell is calculated using time headway. An EV is considered the last vehicle of a cell; hence, the cell clearance time is the travel time of an EV for that cell, which is calculated using the following equation [62]:

(1) $$t_{i}={\overset{˘}{n}}_{i}\times T_{i}$$

Here, $t_{i}$ represents the clearance time of cell *i*, ${\overset{˘}{n}}_{i}$ shows the number of vehicles in *i*, and $T_{i}$ shows the average time headway for *i*.

The average time headway $T_{i}$ can be calculated as [62]:

(2) $$T_{i}=\frac{S_{i}}{\vartheta_{i}}$$

where $S_{i}$ and $\vartheta_{i}$ are the average space headway and the average speed of the vehicles associated with signal *i*, respectively.

Space headway (*S*) [62] can be calculated by using the following equation:

(3) $$S_{i}=\frac{l_{i}}{{\overset{˘}{n}}_{i}}$$

where $l_{i}$ is the length of the cells associated with signal *i* and ${\overset{˘}{n}}_{i}$ is the number of cars in those cells.

The average vehicle speed in cell *i* can be calculated by [63]:

(4) $$\vartheta_{\hat{a_{i}}}=\frac{\sum_{\hat{a_{i}}=1}^{\overset{˘}{n}\hat{a_{i}}}\varkappa_{\hat{a_{i}}}}{{\overset{˘}{n}}_{\hat{a_{i}}}}$$

where $\varkappa_{\hat{a}}$ is the speed of ${\hat{a}}^{th}$ vehicle determined from the relevant speed sensors. If there is no speed sensor installed, $\vartheta_{\hat{a}}$ also can be determined by using the following equation [63]:

(5) $$\vartheta_{\hat{a_{i}}}=\frac{q_{i}}{\rho_{i}}$$

where $q_{i}$ and $\rho_{i}$ represent the flow rate and density associated with *i*th cell, respectively. Density refers to the number of vehicles per unit length of the road. For cell *i*, the density $\rho_{i}$ can be represented as [63]:

(6) $$\rho_{i}=\frac{{\overset{˘}{n}}_{i}}{l_{i}}$$

where ${\overset{˘}{n}}_{i}$ is the number of vehicles that are occupied in length $l_{i}$ of *i*th cell, normally, density is expressed as vehicles per kilometer.

The proposed model calculates the minimum duration a signal needs to be turned green for each EV to cross a cell including the intersection. In this case, a different travel time for an EV to travel from Cell 1 (i.e., dispatch point) to Cell *n* (i.e., incident location) with a different number of signals being turned green is assessed, where *n* is the number of cells between EV dispatch point to the incident location. Please note that a higher number of green signals increases the number of cars waiting in the adjacent areas of EV’s travel path. Our model calculates the wait time of the vehicles in seven cells including the current Cell of an EV, three cells to the right, and three cells to the left of an EV’s travel path as the adjacent areas. To calculate the wait time, we first need to determine when an EV must cross the intersection of those signals, which will be turned green. The time needed to clear the first Cell can be calculated considering the signal is turned to green as:

(7) $$\zeta_{1}=\zeta_{s0}+\zeta_{c1}+\zeta_{\gamma }$$

where $\zeta_{s0}$ is the time taken by an EV to come from the dispatch point to the entry of the first Cell, $\zeta_{c1}$ is the clearance time of cell 1, and $\zeta_{\gamma }$ is the time to clear the intersection. Afterward, the total wait time based on the different number of signals can be calculated as follows:

(8) $$\zeta_{t}=\zeta_{1}+\sum_{j=2}^{m}\zeta_{j},$$

where $\zeta_{t}$ is the EV travel time to clear *m* number of cells plus $\zeta_{1}$, *m* is the number of signals that need to be turned green manually (*m* ranges from 2 to the n−2).

As alluded before, in contrary to previous works [33], the proposed model considers the number of additional vehicles added to the queue in adjacent areas of an EV’s travel path along with its current Cell to calculate the total clearance time. This calculation is also repeated by considering the EV’s current location in other cells (any of the three right and left cells). This provides six additional alternative routes for an EV. If any of the alternate routes are quicker and optimal, the central traffic controller sends the updated route information to the EV and the assistive drones. The number of green signals, which provide less clearance time (EV travel time and the clearance time of observed Cell) is the optimal number of green signals.

The proposed approach also reduces the travel time of an EV in an intersection using drones, which work as the lead vehicle of an EV. Drones will fly to the intersection ahead of an EV to stop any other incoming vehicles and pedestrians. The drones will project the stop sign as shown in Figure 1 and also send audio-visual alert notifications. Drones will assist to keep the intersection clear so that EVs can cross the intersection without slowing down too much to ensure the safety of other road users. Drones will also track the movement of road users near the intersection and instantly notify an EV if there is any sudden unexpected movement from other vehicles or pedestrians approaching the intersection. In this case, drones only need to send a notification if there is sudden movement near the intersection and there is a need for the EV to drive through the intersection cautiously. Otherwise, the EV can safely proceed to and pass the intersection without reducing its speed. Traffic flow and vehicle presence and speed monitoring using UAVs have been extensively studied in the literature [43,45,46]. Our proposed approach can use any of these techniques for objects’ (vehicles and pedestrians) presence and movement detection.

Although drones help EVs to maintain a continuous speed in the proposed approach, there might be some vehicles in the lane ahead of an EV to enter the intersection.

The issue of providing a clearway to an EV near the intersection is addressed using a discrete-time model. This sort of model is used in bilevel optimization trajectory design problems [64]. In this case, the higher level task will be used to clear the intersection for an EV and the lower level task will be assigned to reduce the impact on nonemergency vehicles.

## 4. Performance Evaluation

### 4.1. Simulation Setup

To assess the performance of the proposed model, we used the microscopic version of the open-source, multimodal traffic simulation platform named Simulation of Urban Mobility (SUMO). The Melbourne Central Business District (CBD) map was used in SUMO, where two different incident places were selected. The first incident place was ten signals apart (a short-distance incident afterward) from the EV dispatch location. In comparison, the second incident place was 20 signals apart (termed as a long-distance incident afterward) from the same. For short-distance incidents, we used the EV departure point from St Vincent’s Hospital Melbourne, 41 Victoria Parade, Fitzroy VIC 3065, and the incident location at 64 Smith St, Collingwood VIC 3066. The EV start point for long-distance incidents is similar to short-distance incidents, but the incident location is Bile repair station, 111 Wellington St, Collingwood, VIC 3066. We used five different congestion levels in both scenarios where an occupancy rate of 0% was used to see the travel time in free flow (no other vehicles except EV). A 100% occupancy rate was used to see the impact when the full road segment is occupied, while 30%, 50%, and 70% occupancy rates were used to reflect low, medium, and high congestion, respectively. For low, medium, and high congestion rates, we used real historical data from VicRoads [9]. To reflect different levels of congestion, we selected the average hourly traffic flow data for Saturday from 10:00 a.m. to 11:00 a.m. (low congestion), Monday to Friday from 12:00 p.m. to 01:00 p.m. (medium congestion), and Monday to Friday from 08:00 a.m. to 09:00 a.m. (high congestion). All data were taken as the average of three years’ traffic data for those selected days (e.g., Saturday, Monday–Friday) during the stated time frame.

### 4.2. Simulation Results

We observed flow rate, speed, EV travel time from the dispatch centre to the incident place, and the average waiting time of vehicles in each cell. We used two variations of our proposed model, one with the support of drones (labeled as WD) and the other without the support (labeled as ND), to assess the impact of drone usage. We ran each simulation 20 times, and the average results obtained from them are presented below.

Table 2 and Table 3 show the impact of drone usage and suggest that making more interventions makes an EV reach the incident place faster. For short-distance and a 30% occupancy rate, the EV travel time (without the drone support) reduced from 408, 385, 350, 317, and 295 s to 391, 362, 321, 285, and 267 s, respectively, with the usage of leading drones. A similar pattern can be observed for 50%, 70%, and 100% occupancy rates for both short and long-distance incidents. A lower travel time for EVs with drone support is achieved as drones help EVs to cross the intersection without causing any delay and reducing their speed. The results showed a minimum of 3% lower EV travel time across all cases. Drones can save up to 5 s of EV travel time in each intersection, which plays an important impact in clearing the incident quicker.

Our proposed model aims to reduce EV travel time while ensuring adjacent nonemergency vehicles do not wait longer to provide a giveaway to an EV. We achieve a lower EV travel time by making a dynamic number of interventions (manually turning signals green in front of an EV). Although making more interventions increases the number of vehicles in a cell as it increases the wait time; however, it does not increase the total clearance time. To further explore this issue where total clearance time decreases even when the number of vehicles increases, we monitored density, flow rate, and speed changes, and the results obtained for cell 10 with an occupancy rate of 50% are presented below.

**Figure 4:**
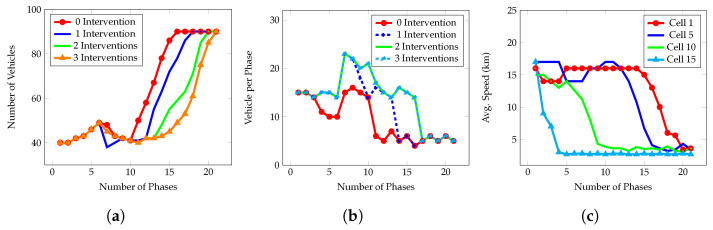
Density, Flow rate, and Speed changes in different cells during the incident with a 50% occupancy rate. (**a**) Density at cell 10. (**b**) Flow rate at Cell 10. (**c**) Speed change at cell 10.

Figure 4a shows that density at Cell 10 started increasing from phase 9 when there was no intervention while the same was observed in phases 12, 15, and 16 for 1, 2, and 3 interventions, respectively.

Figure 4b shows that the flow rate started decreasing mainly from phase 10 with no intervention and phase 13 when 2 interventions were used. With 2 and 3 interventions, the flow rate started decreasing from the 15th and 17th phase, respectively.

Figure 4c shows that speed started decreasing in cell n (where the incident happened) from phase 1 while a similar trend was observed in cells 10, 5, and 1, from phases 5, 11, and 16, respectively. The more vehicles stayed closer to the incident place, the more speed loss was observed.

When an EV reaches the incident place in a shorter time, it brings time headway down. Therefore, even when the number of vehicles increases, the total time to clear the observed cells decreases, as shown in Table 4 and Table 5. Please note that making more interventions does not guarantee a minimum clearance time. When the occupancy rate is too high, making too many signals green will increase the density quickly. For example, when 2 interventions were made with a 70% occupancy rate for the long-distance incident with no drone support, the average cell clearance time was 59.35 s, but it went up to 63.75 s when we made 3 interventions. This happened because when we made 3 interventions, the density of the adjacent cells of the third signal increased too quickly. For the same incident place and occupancy rate, the average cell clearance time was reduced up to 56.45 and 61.15 s, respectively. Overall, for both incident places, the average cell clearance time improvement is around 4.08%. Our simulation also showed that in around 40% cases, our proposed model achieved quicker EV travel time when an alternate route was selected instead of the initial travel path selected at the beginning of the journey.

The proposed model calculates the number of signals that need to be turned green considering a shorter EV travel time and a shorter wait time for surrounding nonemergency vehicles. However, in case of a severe incident, the central traffic controller may only consider the faster travel time of an EV and increase or decrease the number of green signals and finally use the proposed model to calculate the clearance time.

We also compared our proposed model with existing approaches to assess its performance. Table 5 shows the superiority of our approach (UAVES) compared to GreenWave [5] and EPVS [6]. The EV travel time for short-distance incidents with a 50% occupancy rate is 362, 335, and 261 s for GreenWave, EPVS, and UAVES, respectively. On the other hand, the average cell clearance time is 29.25, 24.3, and 23.5 s for GreenWave, EVPS, and UAVES, respectively. Our model improved the EV travel time and average cell clearance time for all other occupancy rates for both incident locations. The use of drones in intersections to provide the EVs with better speed at intersections, selecting an alternate route for EVs based on the updated traffic congestion, and dynamically adjusting the number of green signals resulted in better performance for our proposed model (Table 6).

UAVs have great potential to enhance emergency response times by coordinating with traffic controllers and directing emergency vehicles along optimized paths. However, their use comes with a few constraints, which are discussed below.

i.  Extreme weather conditions: Extreme weather conditions, such as high winds, heavy rain, snow, and foggy visibility conditions may result in communication delay in data transmission between UAVs and control centers and thus impacting its efficacy to provide support. Please note that existing Incident Management System (IMS) of the Intelligent Traffic System (ITS) already incorporates visual roadside units (RSUs), such as speed cameras, red light cameras, and road safety cameras. These RSUs will continue to provide services to EVs if UAVs are unable to provide satisfactory services. Current weather updates can also be taken into consideration to decide whether UAV assistance is to be used.
ii. Battery lifetime: The flight time of the UAVs may differ based on different issues (e.g., the weight of the battery, payload weight, etc.). Existing drone manufacturers provide expected battery life for their products, which is highlighted in Table 1. In our work, we consider that existing drones provided by the manufacturer can be directly integrated into the ITS system without any modifications or the addition of an extra payload. Newer UAVs such as JOUAV CW series VTOL can last up to 480 min and can carry up to 20 kg payload. The price of professional VTOL drones is $3000–300,000 [65]. Depending on the available budget of the transport authority, specific products can be selected based on their requirements (i.e., average estimated flight time to reach the incident location).

## 5. Conclusions

Emergency management systems are one of the most important services provided by governments around the world to ensure public safety and minimise the loss of lives and money. A key task of an EMS is to send the EVs to an incident place faster. Existing literature used priority-based services and changes in traffic signals to ensure faster arrivals of EVs. However, they fail to consider important aspects, such as disruption faced by other nonemergency vehicles, traffic condition changes when the EVs are en route, and speed loss of EVs near the intersections. Our current work addressed these issues and proposed a UAV-assisted adaptive route selection strategy where real-time traffic data obtained from roadside infrastructures and sensors were utilised for a more accurate calculation of traffic parameters and the travels paths were accordingly adjusted to make an EV reach the incident place faster while causing minimal impact on other road users. Simulation results confirmed that our proposed approach outperformed existing methods and achieved a lower EV arrival time and cell clearance time.

## Figures and Tables

**Table 1:** Drone specification.

Name of Drone	Flying Time	Communication Frequency (GHz)	Communication Range	Max Flying Speed	Price (USD)
[49]	30 min (at 20 kph, no wind)	2.400–2.483 5.725–5.825	Up to 7 km	72 km/h	1599
[50]	31 min (at 25 kph, no wind)	2.400–2.483 5.725–5.850	Up to 8 km	72 km/h	1599
[51]	27 min (hovering at sea level, no wind)	2.400–2.483 5.725–5.850	Up to 7 km	94 km/h	3299
[52]	40 min	2.400–2.4835	Up to 9 km	72 km/h	1495
[53]	23 min	2.4–2.483 5.18–5.24 5.725-5.85	Up to 3.5 km	58 km/h	999
[54]	25 min	2.4–2.48 5.2–5.8	Up to 800 m	29 km/h	599
[55]	25 min	2.4–2.48 5.2–5.8	Up to 4 km	55 km/h	999
[56]	35 min	5.725–5.850	Up to 8 km	64 km/h	499
[57]	17 min	2.405–2.475	Up to 800 m	65 km/h	987
[58]	25 min	2.402–2.476 5.727–5.8	Up to 2 km	57 km/h	799

**Table 2:** EV Travel time (s) for different occupancy rates with and without drone support for a short distance.

Intervention (IN)	Occupancy Rate (%)									
	0		30		50		70		100	
	ND	WD	ND	WD	ND	WD	ND	WD	ND	WD
0	230	213	408	385	515	478	710	645	2392	2184
1	230	213	385	362	464	432	682	634	3275	3107
2	230	213	350	321	403	357	621	558	3221	3095
3	230	213	317	285	371	339	587	503	3180	2984
4	230	213	295	267	342	323	509	482	2886	2712

ND = No drone support, WD = with drone support.

**Table 3:** EV Travel time (s) for different occupancy rates with and without drone support for long distance.

Intervention (IN)	Occupancy Rate (%)									
	0		30		50		70		100	
	ND	WD	ND	WD	ND	WD	ND	WD	ND	WD
0	480	462	811	787	981	925	1323	1289	3843	3762
1	480	457	745	721	917	891	1288	1231	3819	3701
2	480	453	727	675	878	851	1216	1192	3902	3684
3	480	448	682	623	829	801	1188	1152	3874	3672
4	480	443	615	578	787	765	1102	1092	3716	3658

ND = No drone support, WD = with drone support.

**Table 4:** Average cell clearance time (s) for short-distance incident (distance = ten signals).

S #1	Interventions										
	0	1	1	2	2	3	3	4	4	5	5
**O (%)**	**ND**	**ND**	**WD**	**ND**	**WD**	**ND**	**WD**	**ND**	**WD**	**ND**	**WD**
**0**	23.1	23.1	23.1	23.1	23.1	23.1	23.1	23.1	23.1	23.1	**23.1**
**30**	29.25	28.6	28.2	26.0	25.1	24.3	23.5	26.1	31.9	35.1	39.36
**50**	31.15	29.7	28.6	26.4	**25.9**	31.9	29.6	37.25	34.7	44.2	40.6
**70**	51.15	48.1	**46.75**	48.4	44.45	56.85	49.9	61.9	56.05	69.45	62.55
**100**	**130.9**	144.85	133.55	179.85	165.6	192.45	178.8	206.4	186.35	229.85	205.6

ND = No drone support, WD = with drone support, O = Occupancy rate.

**Table 5:** Cell clearance time (s) for long-distance incident (distance = 20 signals).

S #2	Interventions										
	0	1	1	2	2	3	3	4	4	5	5
**O (%)**	**ND**	**ND**	**WD**	**ND**	**WD**	**ND**	**WD**	**ND**	**WD**	**ND**	**WD**
**0**	24	24	24	24	24	24	24	24	24	24	**24**
**30**	36.25	34.71	33.6	31.2	29.75	29.90	**28.65**	36.75	35.2	44.35	42.9
**50**	43.75	42.1	41.25	38.7	**36.75**	46.25	44.65	50.7	48.75	62.7	59.75
**70**	56.15	55.2	**53.6**	59.35	56.45	63.75	61.15	66.75	65.6	72.6	69.15
**100**	**188.1**	217.4	207.65	256.15	240.2	266.15	262.1	275.65	271.2	285.8	281.35

ND = No drone support, WD = with drone support, O = Occupancy rate.

**Table 6:** Comparison between Green Wave, EVPS, and UAVES.

	Occupancy	Green Wave		EVPS		UAVES	
		EV Travel Time (s)	Avg. Cell Clearance Time (s)	EV Travel Time (s)	Avg. Cell Clearance Time (s)	EV Travel Time (s)	Avg. Cell Clearance Time (s)
Short Distance	30	331	29.25	289	24.3	276	23.5
	50	362	31.15	335	29.6	261	25.9
	70	573	51.27	512	48.4	487	44.45
Long Distance	30	806	36.25	675	29.90	635	28.65
	50	915	43.75	854	38.7	823	36.75
	70	1225	56.15	1146	55.2	1102	53.5

## Data Availability

Publicly available datasets were analyzed in this study. This data can be found here: [https://vicroadsopendata-vicroadsmaps.opendata.arcgis.com/, accessed on 28 May 2023].
